# Characteristics and risk factors for colorectal polyps among children in an urban area of Wenzhou, China: a retrospective case control study

**DOI:** 10.1186/s12887-023-04197-6

**Published:** 2023-08-19

**Authors:** Yinghui Wang, Lingjuan Fang, Kaiyu Huang, Tongtong Pan, Huajun Lu, Xiumei Yan

**Affiliations:** Department of Pediatric Gastroenterology, The Second Affiliated Hospital and Yuying Children’s Hospital of Wenzhou Medical University, NO. 109 Xueyuan Road, Wenzhou, 325000 Zhejiang Province China

**Keywords:** Colorectal polyps, Children, Risk factors, Characteristics

## Abstract

**Background:**

Scarce evidence exists on pediatric colorectal polyp risk factors. This study explored the clinical manifestations, morphological and pathological characteristics of, and risk factors for pediatric colorectal polyps.

**Methods:**

This retrospective case-control study included children who received colonoscopy, divided into a colorectal polyp group and a normal control group based on colonoscopy results. The risk factors for colorectal polyps in children were analyzed through logistic regression analysis.

**Results:**

The mean age of children with polyps was 6.77 ± 3.44 years. Polyps were detected predominantly in males (72.9%); hematochezia was the primary clinical manifestation (80.25%). Most polyps were juvenile (88.9%) and solitary (87.7%); 50.6% were located in the rectosigmoid area. Univariate analysis showed that gender (*P* = 0.037), age (*P* < 0.001), family aggregation (*P* < 0.001), specific immunoglobulin E (sIgE) (*P* < 0.001), platelet count (P = 0.001), aspartate aminotransferase (AST) (P = 0.016), meat intake (P = 0.010), and vegetable intake (P < 0.001) were significantly associated with colorectal polyps. Age ≤ 6 years (3–6 years: OR: 26.601, 95% CI: 3.761–160.910; < 3 years: OR: 22.678, 95% CI: 1.873–274.535), positive family aggregation (OR: 3.540, 95% CI: 1.177–10.643), positive sIgE (OR:2.263, 95% CI: 1.076–4.761), and higher meat intake (OR:1.046, 95% CI: 1.029–1.063) were risk factors for pediatric colorectal polyps in logistic regression analysis. Higher vegetable intake (OR: 0.993, 95% CI: 0.986–1.000) was a protective factor against pediatric colorectal polyps. The area under the curve (AUC) of meat intake in the receiver operating characteristic (ROC) curve analysis for predicting colorectal polyps was 0.607; the best cut-off value was 92.14 g/d (*P* = 0.010, 95% CI: 0.527–0.687). The meat and vegetable intake combination AUC in predicting pediatric colorectal polyps was 0.781 (*P* < 0.001, 95% CI: 0.718–0.845).

**Conclusions:**

Juvenile, solitary, and located in the rectosigmoid region polyps are most common in children. Hematochezia is the main clinical manifestation. Most polyps were, but multiple and proximally located polyps were also detected. Age ≤ 6 years, especially 3–6 years, positive family aggregation, positive sIgE, and higher meat intake are risk factors for pediatric colorectal polyps. A higher vegetable intake is a protective factor.

## Background

Colorectal polyps consist of colonic mucosa that rises from the mucous membrane to the bowel lumen [1]. Most children with polyps have long-term painless, benign blood stools, although some have massive gastrointestinal bleeding, causing hemorrhagic shock [1]. According to their pathological type, colorectal polyps can be divided into juvenile, inflammatory, adenomatous, and hyperplastic polyps. According to the theory “polyp–adenoma–cancer” [2], adenomatous polyp is an important part of the development of colorectal cancer. Most colorectal polyps in children are benign juvenile polyps, which, however, have the potential to become malignant [3, 4]. Early detection and colonoscopic removal of colorectal polyps is essential as they can lead to intestinal obstruction, anemia, hemorrhagic shock, and carcinogenesis. However, due to its invasiveness, colonoscopy has not been widely applied in children. Thus, exploring the risk factors for colorectal polyps may be much more effective for disease prevention. Studies in adults have found that the presence of colorectal polyps is closely related to age, weight [5], diet [6, 7], smoking [8], and other factors, but there are few studies on the risk factors for colorectal polyps in children. Therefore, we collected the clinical data of children undergoing colonoscopy and retrospectively analyzed the clinical manifestations and colonoscopic and pathological features of colorectal polyps in children, as well as the risk factors for pediatric colorectal polyps.

## Methods

### Participants

A total number of 81 children who were hospitalized from October 2020 to October 2022 and diagnosed with colorectal polyps by colonoscopy were enrolled as a case group. In the control group, we randomly selected and included 123 children who underwent colonoscopy during the same period and whose results showed no obvious abnormalities. If a patient had undergone multiple colonoscopies within the time frame selected, only the first colonoscopy was included in our research work to ensure per-patient analysis results have been obtained.

The following inclusion criteria were implemented: (1) Children aged 0–18 years who underwent colonoscopy; (2) Complete clinical data, including clear image data of colonoscopy and polyp histopathology; (3) Informed consent was acquired, and the respective forms were signed by the patient’s parents, including informed consent for colonoscopy, informed consent for biopsy tissue, and informed consent for endoscopic polypectomy.

The exclusion criteria applied were as follows: (1) Severe cardiopulmonary disease or other serious underlying diseases; (2) Acute active stage of intestinal inflammatory diseases; (3) Coagulation dysfunction, bleeding tendency; (4) Severe spinal deformity; (5) Poor compliance. Colonoscopy is not recommended in children with these conditions.

### Ethics approval and consent to participate

This clinical study was approved by the Ethics Committee of The Second Affiliated Hospital and Yuying Children’s Hospital of Wenzhou Medical University (Wenzhou, Zhejiang, China). All methods and procedures were performed in accordance with the ethical standards as laid down in the Declaration of Helsinki. Written informed consent was obtained from all participants’ legal guardian.

### Data collection

Clinical data were collected from electronic and paper medical records, including demographic characteristics and clinical manifestations. The residential addresses were divided into urban and rural. Based on clinical significance, we converted continuous data into categorical data. Age was classified into four categories:< 3 years, 3–6 years, 6–11 years, and ≥ 12 years; the course of disease was divided into a short course (< 6 months) and a long course (≥ 6 months).

The following blood test data were collected: hemoglobin (venous blood), sIgE, the white blood cell (WBC) count, platelet count, blood eosinophil count, C-reactive protein (CRP), albumin, alanine aminotransferase (ALT), AST, alkaline phosphatase (ALP), and gamma-glutamyl transferase (γ-GT). Based on the criteria of the World Health Organization (2011) [9], anemia (Hb < 110 g/L) in the age group 6–59 months was defined as mild (100–109 g/L), moderate (70–99 g/L), and severe (< 70 g/L). In the 5–11-year age group, anemia (Hb < 115 g/L) was classified into mild (110–114 g/L), moderate (80–109 g/L), and severe (< 80 g/L). Anemia (Hb < 120 g/L) in the age group of 12–14 years was categorized into mild (110–119 g/L), moderate (80–109 g/L), and severe (< 80 g/L).Based on current international standards, a concentration of sIgE < 0.35 kU/L was considered to indicate a negative result; otherwise, the result was considered positive [10].

Information was obtained by asking parents about their children’s defecation habits, dietary habits, and family aggregation. Diarrhea was considered to be present if their defecation habits met the Rome IV diagnostic criteria for diarrhea [11]. Similarly, we considered their defecation habits as constipation if they met the Rome IV diagnostic criteria for constipation [11]. We collected patients’ diet data through 3-day 24-hour dietary recall. We examined the consumption of three main food groups: meat (pork, chicken, duck, etc.), vegetables (green vegetables, cabbage, tomato, lettuce, etc.), and seafood (fish, shrimp, shell, etc.). The estimates of intake (g/day) were based on frequency and portion size information. If a relative of the patient had polyps or bowel cancer, it was considered that family aggregation is positive.

For the colonoscopy report of each patient, we extracted the following results: quality of intestinal preparation, depth and location of colonoscopy, presence or absence of polyps, location and characterizations of the polyps (such as number, size, and shape), and the pathologic type of the polyps (juvenile polyps/adenomatous polyps/hyperplastic polyps/inflammatory polyps/juvenile polyposis). To facilitate statistical data collection and analysis, the locations of polyps were grouped into rectal polyps, sigmoid polyps, descending colon polyps, pan-colorectal polyps, and polyps in other locations (including ileocecal, ascending, and transverse colon polyps), the number of polyps was grouped into solitary polyp (only one polyp) and multiple polyps (containing at least two polyps), the size of polyps was categorized into large polyps (maximum diameter of the polyps > 15 mm) and small polyps (maximum diameter of the polyps ≤ 15 mm). The shapes of polyps were grouped into sessile, pedunculated- sessile, and pedunculated polyps.

### Statistical analysis

All statistical analyses were conducted using SPSS 22.0. Continuous variables were presented as mean with standard vision or medians with interquartile ranges. The difference between groups was compared by t-test or nonparametric test. Categorical variables were expressed as frequencies with percentages, and the differences between groups were tested by χ2 tests or Fisher’s exact tests and considerable covariates (*P* < 0.10) were included in multiple analyses conducted using logistic regression. A *P* < 0.05 (two tailed) was considered statistically significant. ROC curve analysis was used to evaluate the predictive ability and optimal cut-off values of statistically significant continuous variables.

## Results

### Characteristics of the colorectal polyp group and the normal control group

A total number of 204 patients were enrolled in the study, of whom 81 had colorectal polyps. The mean age of the children with polyps was 6.77 ± 3.44 years, and the mean age of children without polyps was 9.27 ± 3.02 years. Compared with the normal control group, the patients who had colorectal polyps tended to have the following characteristics: male, younger, with anemia, with positive family aggregation and sIgE, with a higher platelet count and AST level, and with a higher intake of meat but a lower intake of vegetable (Table 1). There was no significant difference in the defecation habits, type of residence, WBC count, blood eosinophil count, CRP, albumin, ALT, ALP, γ-GT, and seafood intake (*P* > 0.05).

**Table 1:** Characteristics of the colorectal polyp group and the normal control group

Variable			Without colorectal polyp (n = 123)			With colorectal polyp (n = 81)	*P*-value
Sex (%)							
Male			72 (58.5%)			59 (72.8%)	0.037
Female			51 (41.5%)			22 (27.2%)	
Age, y < 3 3–6 6–11 ≥12			9.27 ± 3.02 6 (4.9%) 22 (17.9%) 66 (53.7%) 29 (23.6%)			6.77 ± 3.44 7 (8.6%) 31 (38.3%) 38 (46.9%) 5 (6.2%)	< 0.001
Family aggregation (%)							
Negative			113 (92.6%)			58 (71.6%)	< 0.001
Positive			9 (7.4%)			23 (28.4%)	
Defecation habits (%)							
Normal			74 (60.2%)			51 (63.0%)	0.893
Constipation			38 (30.9%)			24 (29.6%)	
Diarrhea			11 (8.9%)			6 (7.4%)	
Type of residence (%)							
Town			92 (74.8%)			66 (81.5%)	0.264
Country			31 (25.2%)			15 (18.5%)	
Specific IgE (%)							
Negative			78 (63.4%)			30 (37.0%)	< 0.001
Positive			45 (36.3%)			51 (63.0%)	
Hemoglobin (%)							
Normal			110 (89.4%)			63 (77.8%)	0.011
Mild anemia			7 (5.7%)			9 (11.1%)	
Moderate anemia			3 (2.4%)			9 (11.1%)	
Severe anemia			3 (2.4%)			0 (0.0%)	
White blood cell count (×10^9^/L)			6.29 (2.22)			6.62 (3.06)	0.286
Platelet count (×10^9^/L)			182.0 (96.0)			214.0 (78.0)	0.001
Blood eosinophil count (×10^9^/L)			0.13 (0.11)			0.12 (0.17)	0.595
C-reactive protein (mg/L)			0.50 (0.80)			0.68 (1.36)	0.079
Albumin (g/L)			46.30 ± 2.76			45.60 ± 2.41	0.067
Alanine aminotransferase (U/L)			14.0 (7.3)			14.5 (6.0)	0.448
Aspartate aminotransferase (U/L)			29.0 (9.0)			31.0 (13.0)	0.016
Alkaline phosphatase (U/L)			221.0 (83.0)			205.0 (62.0)	0.236
Gamma-glutamyl transferase (U/L)			12.0 (6.0)			10.0 (7.0)	0.238
Meat intake (g/d)			71.43 (46.43)			95.0 (70.54)	0.010
Vegetable intake (g/d)			250.0 (100.0)			200.0 (150.0)	< 0.001
Seafood intake (g/d)			50.0 (25.0)			42.86 (14.29)	0.152

### Correlation analysis of clinical manifestations in patients with polyps

Children with colorectal polyps had different clinical manifestations, with incidences of clinical manifestations from high to low as follows: hematochezia (80.2%), abdominal pain (23.5%), and anal mass prolapse (8.6%). There were also a few patients without obvious clinical manifestations (6.2%) (including imaging suggesting the presence of polyps, CA199 found elevated, etc.). Long-course disease was observed in 67.9% of the cases, whereas short-course disease accounted for 32.1%. Comparing the incidence of each clinical manifestation in different age groups, the differences in abdominal pain was statistically significant (*P* = 0.007), whereas the differences in the remaining clinical manifestations were not statistically significant (*P* > 0.05) (Table 2). Comparing the incidence of various clinical manifestations in different gender groups, the difference in abdominal pain was statistically significant (*P* = 0.024), whereas the differences in the incidence of the remaining clinical manifestations were not statistically significant (*P* > 0.05) (Table 2).

**Table 2:** The correlation analysis of clinical manifestations in patients with polyps

Group	Hematochezia		Abdominal pain		Anal mass prolapse	
	No (%)	Yes (%)	No (%)	Yes (%)	No (%)	Yes (%)
Age, y						
< 3	1 (14.3%)	6 (85.7%)	7 (100.0%)	0 (0.0%)	5 (71.4%)	2 (28.6%)
3–6	4 (12.9%)	27 (87.1%)	28 (90.3%)	3 (9.7%)	27 (87.1%)	4 (12.9%)
6–11	9 (23.7%)	29 (76.3%)	25 (65.8%)	13 (34.2%)	37 (97.4%)	1 (2.6%)
≥12	2 (40.0%)	3 (60.0%)	2 (40.0%)	3 (60.0%)	5 (4.6%)	0 (0.0%)
χ2	2.857		10.877		5.732	
*P*-value	0.392		0.007		0.091	
Sex						
Male	12 (20.3%))	47 (79.7%)	49 (83.1%)	10 (16.9%)	53 (89.8%)	6 (10.2%)
Female	4 (18.2%)	18 (81.8%)	13 (59.1%)	9 (40.9%))	21 (95.5%)	1 (4.5%)
χ2	0.047		5.124		0.642	
*P*-value	0.828		0.024		0.423	

### Morphological and pathological characteristics of colorectal polyps

The main features of colorectal polyps are presented in Table 3. The pathological type of polyps was predominantly juvenile (n = 72, 88.9%). Adenomatous polyps were found in five cases (6.2%), 4 of which were associated with low-grade intraepithelial neoplasia. There were two cases of inflammatory polyps (2.5%), one of which with adenomatous gland hyperplasia. Hyperplastic polyps were established in one case (1.2%) and juvenile polyposis in one case (1.2%). The majority of children (n = 71, 87.7%) had only one polyp, but in 10 cases (12.3%), multiple polyps were present. Small polyps were detected in 47 cases (58.0%), whereas big polyps were present in 31 cases (38.3%). Polyps were located mainly in the rectum (n = 41, 50.6%) and the sigmoid colon (n = 19, 23.5%). Descending colon polyps were found in 7 cases (8.6%); polyps in other locations of the colon (including ileocecal, ascending, and transverse colon) were detected in 10 cases (12.3%). In 4 cases (4.9%), the polyps were distributed in various parts of the colon. The majority of polyps were pedunculated (n = 51, 62.96%), followed by pedunculated-sessile (n = 22, 27.16%); sessile polyps were established in 6 cases (7.41%).

**Table 3:** Main characteristics of colorectal polyps in children

Characteristics (n = 81)	N (%)
Pathological type	
Juvenile polyps	72 (88.9%)
Adenomatous polyps	5 (6.2%)
Inflammatory polyps	2 (2.5%)
Hyperplastic polyp Juvenile polyposis	1 (1.2%) 1 (1.2%)
Number	
Single	71 (87.7%)
Multiple	10 (12.3%)
Size	
Small	47 (58.0%)
Big	31 (38.3%)
All have	3 (3.7%)
Location	
Rectum	41 (50.6%)
Sigmoid colon	19 (23.5%)
Descending colon	7 (8.6%)
Pan-colon	4 (4.9%)
Other locations	10 (12.3%)
Type	
Pedunculated	51 (62.9%)
Pedunculated-sessile	22 (27.2%)
Sessile	6 (7.4%)
All have	2 (2.5%)

### Risk factors associated with the presence of colorectal polyps

The potential influencing factors of colorectal polyps in children are listed in Table 4. Univariate analysis showed that sex, age, family aggregation, sIgE, platelet count, AST, and meat and vegetable intake were associated with the presence of colorectal polyps in children (*P* < 0.05). In contrast, type of residence, defecation habits, the WBC count, blood eosinophil count, CRP, albumin, ALT, ALP, γ-GT, and seafood intake were not associated with colorectal polyps (*P* > 0.05).

**Table 4:** Univariate analysis of the factors for the presence of colorectal polyps in children

Variable			Without colorectal polyp (n = 123)			With colorectal polyp (n = 81)	*P*-value
Sex (%)							
Male			72 (58.5%)			59 (72.8%)	0.037
Female			51 (41.5%)			22 (27.2%)	
Age, y < 3 3–6 6–11 ≥12			6 (4.9%) 22 (17.9%) 66 (53.7%) 29 (23.6%)			7 (8.6%) 31 (38.3%) 38 (46.9%) 5 (6.2%)	< 0.001
Family aggregation (%)							
Negative			113 (92.6%)			58 (71.6%)	< 0.001
Positive			9 (7.4%)			23 (28.4%)	
Defecation habits (%)							
Normal			74 (60.2%)			51 (63.0%)	0.893
Constipation			38 (30.9%)			24 (29.6%)	
Diarrhea			11 (8.9%)			6 (7.4%)	
Type of residence (%)							
Town			92 (74.8%)			66 (81.5%)	0.264
Country			31 (25.2%)			15 (18.5%)	
Specific IgE (%)							
Negative			78 (63.4%)			30 (37.0%)	< 0.001
Positive			45 (36.3%)			51 (63.0%)	
White blood cell count (×10^9^/L)			6.29 (2.22)			6.62 (3.06)	0.286
Platelet count (×10^9^/L)			182.0 (96.0)			214.0 (78.0)	0.001
Blood eosinophil count (×10^9^/L)			0.13 (0.11)			0.12 (0.17)	0.595
C-reactive protein (mg/L)			0.50 (0.80)			0.68 (1.36)	0.079
Albumin (g/L)			46.30 ± 2.76			45.60 ± 2.41	0.067
Alanine aminotransferase (U/L)			14.0 (7.3)			14.5 (6.0)	0.448
Aspartate aminotransferase (U/L)			29.0 (9.0)			31.0 (13.0)	0.016
Alkaline phosphatase (U/L)			221.0 (83.0)			205.0 (62.0)	0.236
Gamma-glutamyl transferase (U/L)			12.0 (6.0)			10.0 (7.0)	0.238
Meat intake (g/d)			71.43 (46.43)			95.0 (70.54)	0.010
Vegetable intake (g/d)			250.0 (100.0)			200.0 (150.0)	< 0.001
Seafood intake (g/d)			50.0 (25.0)			42.86 (14.29)	0.152

**Fig. 1:**
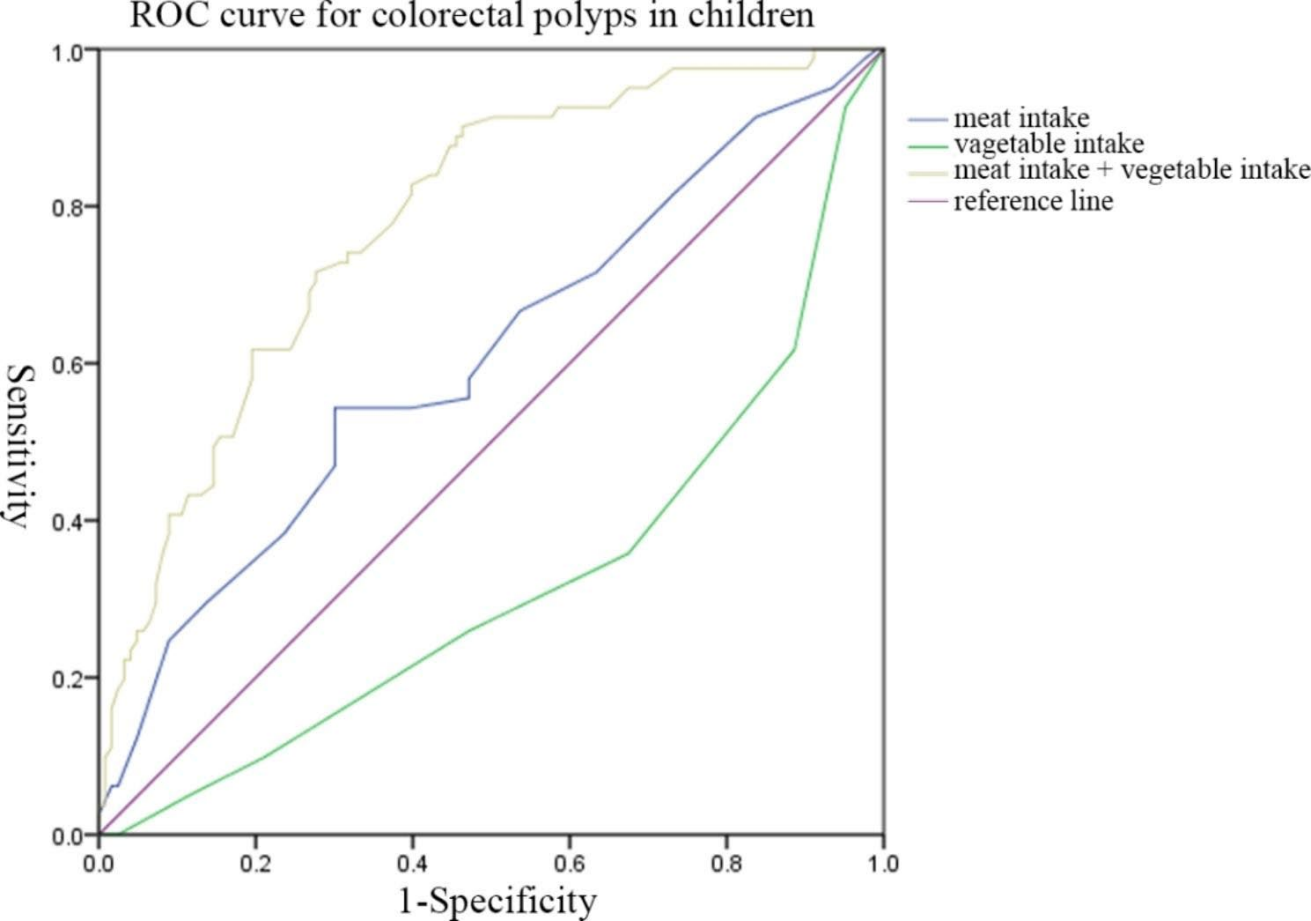
the Receiver operating characteristics (ROC) curve of meat intake, vegetable intake, combination of meat intake, and vegetable intake for colorectal polyps in children

Considerable covariates (*P* < 0.10 or with important clinical relevance) were included in the logistic regression analysis. Multivariate analysis revealed that age ≤ 6 years, positive family aggregation, positive sIgE, and a higher intake of meat were risk factors for the presence of colorectal polyps in children. Meanwhile, a higher intake of vegetable was a protective factor for colorectal polyps in children. Compared to children who aged > 6 years, children with onset age at 6 or younger had a higher risk for colorectal polyps (OR:22.678,95% CI: 1.873–274.535) (OR:24.601,95% CI: 3.761–160.910) and the risk of colorectal polyps is highest in children aged 3–6 years. Children with positive family aggregation had a higher risk for colorectal polyps than those with negative family aggregation (OR:3.540,95% CI: 1.177–10.643). Children with positive sIgE had a higher risk for colorectal polyps than those with negative sIgE (OR:2.263,95% CI: 1.076–4.761). Children with higher intake of meat had a higher risk for colorectal polyps (OR:1.046,95% CI: 1.029–1.063) and those with higher intake of vegetable had a lower risk for colorectal polyps (OR:0.993,95% CI: 0.986–1.000) (Table 5). The predictive value of meat intake, vegetable intake, combination of meat intake and vegetable intake on colorectal polyps in children was analyzed by the ROC curve analysis (Fig. 1). The AUC of meat intake for colorectal polyps in children was 0.607, which had predictive value. The optimal cut-off value in this study was 92.14 g/d (*P* = 0.010,95% CI: 0.527–0.687) and the sensitivity and specificity were 66.67% and 69.92%, respectively. The AUC of vegetable intake for predicting colorectal polyps in children was 0.319, and the predictive value alone was low. The AUC of the combination of meat intake and vegetable intake for predicting colorectal polyps in children was 0.781. The predictive value was better than that of meat intake alone (*P* < 0.001,95% CI: 0.718–0.845).

**Table 5:** Multivariate analysis of the factors for the presence of the colorectal polyps in children

Variable	β	OR	*P*-value	Lower limit of 95%	Upper limit of 95%
Sex (%)					
Male		1.000			
Female	-0.329	0.719	0.418	0.324	1.596
Age, y (%)					
< 3	3.121	22.678	0.014	1.873	274.535
3–6	3.203	24.601	0.001	3.761	160.910
6–11	0.931	2.537	0.197	0.616	10.448
≥12		1.000			
Family aggregation (%)					
Negative		1.000			
Positive	1.264	3.540	0.024	1.177	10.643
Specific IgE (%)					
Negative		1.000			
Positive	0.817	2.263	0.031	1.076	4.761
Albumin (g/L)	-0.027	0.973	0.729	0.835	1.135
Platelet count (×10^9^/L)	0.005	1.005	0.059	1.000	1.011
C-reactive protein (mg/L)	0.013	1.013	0.432	0.981	1.046
Aspartate aminotransferase (U/L)	0.006	1.006	0.771	0.966	1.047
Meat intake (g/d)	0.045	1.046	< 0.001	1.029	1.063
Vegetable intake (g/d)	-0.007	0.993	0.041	0.986	1.000

## Discussion

Colorectal polyps are the most frequently reported cause of lower gastrointestinal bleeding in children [12]. In our study, hematochezia was the most common presentation, which is consistent with other studies [13–16], and abdominal pain and anal mass prolapse are also other relatively infrequent clinical manifestations. Yang et al. [17] found no significant differences in the clinical manifestations in different gender and age groups. However, we established that abdominal pain was associated with age and gender. This may be due to the different age grouping criteria. The intestine is innervated mainly by sympathetic, parasympathetic, and autonomic nerves, and is sensitive to distraction, temperature, and chemical stimulations. Some polyps in the upper colon may affect the movement of the intestine, causing abdominal pain due to tensile irritation of the intestines [13]. In our study, children older than six years were more likely to manifest as abdominal pain, which may be due to the fact that younger children cannot accurately express abdominal pain. Females were more likely to have manifestations such as abdominal pain, which has not been previously reported.

Our study showed that polyps were mainly solitary and located in the rectosigmoid region, which is generally consistent with the available clinical analysis data [15, 16, 18, 19]. Although most polyps were located in rectosigmoid region, the incidence of the proximal location varied from 4.2 to 35% [3, 14, 15, 20]. In our study, there were 12.3% of polyps located in other locations of colon (including ileocecal, ascending, and transverse colon polyps), and 4.9% of polyps in the pan-colon. Meanwhile, the incidence of multiple polyps varied from 11.6 to 39.1% [19–21], and 12.3% of patients had multiple polyps in our study. Thus, a total colonoscopy is recommended for children with suspected colorectal polyps.

Juvenile polyps were the most common pathological type of pediatric colorectal polyps in this study. Juvenile polyps were present in 88.9% of the cases, followed by adenomatous polyps, inflammatory polyps, hyperplastic polyps, and juvenile polyposis, which accounted for 6.2%, 2.5%, 1.2%, and 1.2%, respectively. Previously published data for Hongkong [22] showed similar incidences of juvenile and adenomatous polyps. In an Iranian study [15], lower incidences of inflammatory and adenomatous polyps were reported, but the incidences of juvenile and hyperplastic polyps were similar to those established in the present study. The incidence of juvenile polyposis in our research work was lower than those in other reports [15, 23, 24], which was possibly because colonoscopy was not performed in all first-degree relatives. Haghi et al. [15] found adenomatous transformation in 3% of juvenile polyposis and Gupta et al. [3] reported a case of juvenile polyp with adenomatous changes and adenocarcinoma in situ. However, in our study, no juvenile polyps/juvenile polyposis with adenomatous changes was found. Thus, we consider that adenomatous changes and adenocarcinoma are uncommon in juvenile polyps/juvenile polyposis. In this study, one case of inflammatory polyps with a few adenomatous hyperplasia glands was established. Inflammatory polyps may undergo carcinogenesis, and hyperplastic polyps containing adenomatous foci may also become malignant, which is a risk factor for colon cancer [25, 26]. Adenomatous polyps are recognized as precancerous lesions of colon cancer [2]. There were four cases of adenomatous polyps associated with low-grade intraepithelial neoplasia in our study. However, the evolution of adenomatous polyps progresses over a long time. Therefore, regular colonoscopy at follow-up examinations is recommended for children with adenomatous polyps.

The incidence of colorectal polyps has been on the increase recently, which may be related to the improvements in equipment or colonoscopy techniques that increase the detection of colorectal polyps, as well as changes in diet and lifestyle.

As modifiable risk factors, studies on adults suggest that the presence of colorectal polyps may be closely related to weight [27, 28], diet [6, 29, 30], smoking [8, 31, 32], and other factors. Our study also identified a higher intake of meat as a risk factor for colorectal polyps, whereas a higher intake of vegetable served as a protective factor for colorectal polyps in children. The risk of high meat intake may be related to the production of carcinogenic substances such as heterocyclic amines and polycyclic aromatic hydrocarbons in meat in the process of high-temperature cooking [7]. The specific mechanism needs to be further explored. A meta-analysis [6] showed that individuals who have a high consumption of red and processed meats are at a 22% more risk of developing adenomatous polyps compared to individuals who have a low intake of red and processed meat. Our analysis in this study established that the optimal cut-off value of meat intake was 92.14 g/d, but as the differences in the dietary culture of different regions and the sample size were not large, this data can be used as a reference. Epidemiological studies on vegetables have obtained conflicting results [29, 33, 34]. Kunzmann et al. [29] showed that the increasing intake of vegetables can prevent the development of multiple adenomas and may reduce the adverse effects of high-processed meat intake on the risk of colorectal cancer. Godos et al. [34] defined a healthy dietary pattern as a diet high in fruits and vegetables and an unhealthy diet as a diet high in red meat/processed meat, salt, sugar, and refined grains. The study results showed that individuals who adhered to healthy dietary pattern had a reduced risk of colorectal adenomas by nearly 20%, whereas individuals with unhealthy dietary patterns had an increased relative risk of colorectal adenomas by nearly 25%.

Studies have found that children with colorectal polyps usually have changes in defecation [14, 35, 36]. The study [37] on adults showed that the prevalence and incidence of colorectal cancer and colorectal polyps were significantly higher in patients with chronic constipation, the risk of which increased with the severity of chronic constipation. It was also proposed that constipation may be an influential factor of colon polyps in children [17]. According to the Rome IV diagnostic criteria for diarrhea [11], defecation behaviors with changes in stool traits, such as loose stool and pasty stool, accounted for more than 25% of all defecation behaviors, we considered their defecation habits to be diarrhea in our study. In this study, there was no statistically significant difference in defecation habits between the colorectal polyp group and the normal control group. Probably because the subjects were hospitalized children in the pediatric gastroenterology department and had a higher incidence of abnormal defecation habits than out-of-hospital children. For children who usually have abnormal defecation habits, we recommend them for active prevention and treatment.

Alexand et al. [38] found a large amount of eosinophil infiltration in juvenile polyp specimens and first suggested that juvenile polyps may be associated with food allergies. Huan et al. [39] and Yang et al. [17] also proposed that allergy may increase the risk of developing colorectal polyps in children by retrospectively analyzing clinical data. In our study, we collected sIgE and also found statistically significant differences between the colorectal polyp group and the normal control group, which further suggested the association between allergy and colorectal polyps. It may be because when the body is in an allergic state, chronic inflammation will promote granulation hyperplasia after mechanical damage stimulation of hard feces, and then form protuberant polyps. The diagnosis of allergy also needs to be combined with clinical manifestations, which is also a deficiency in our collection process. The association between allergy and polyps still needs further study.

It is widely mentioned the association between polyposis syndrome and family aggregation [14, 19, 40]. The relationship between family aggregation and colorectal polyps in children with non-polyposis syndrome has rarely been discussed. In our study, the majority of patients were non-polyposis syndrome and we found that positive family aggregation was a risk factor for colorectal polyps. It is probably because children usually have eating and living habits similar to those of their parents; additionally, genetic factors may be associated with colorectal polyps’ incidence. Studies [41] have revealed that first-degree relatives of patients with colorectal polyps still have a high risk of colorectal cancer, so early screening is needed.

Age may be an immutable factor for colorectal polyps. Our study found that age ≤ 6 years, especially 3–6 years, was a risk factor for colorectal polyps, which is in agreement with the findings of domestic and foreign data [22, 42]. The rapid intestinal development in preschool children, under the action of various hormones in the body, leads to excessive proliferation of intestinal mucosa resulting in the formation of polyps [35], while self-dislodgement of colorectal polyps may occur in older children [43].

In the univariate analysis of colorectal polyps, we found that gender was associated with the presence of colorectal polyps, but in multivariate analysis, gender was not a risk factor for polyps. There may be some bias related to the fact that our overall sample was more males dominated. It was reported that the incidence of colorectal polyps is higher in male than in female [12, 44], which may be related to the facts that estrogen is a protective factor for the intestines [45] and the presence of bad habits such as alcohol consumption, smoking and overeating in adult men [46], but children do not have such bad habits. The relation between age and polyps needs to be further analyzed.

There are some limitations in this study. First, this was a retrospective single-center observational study. Second, eating and defecation habits were self-reported by parents and there may have some recall bias. Finally, the number of cases included in this study is not large, and other risk factors that may be associated with the presence of colorectal polyps in children remain to be explored. The association and predictive value of these factors with colorectal polyps in children cannot be fully determined based on the results of this study alone, and the findings need to be validated in a multicenter study with a large sample.

## Conclusions

In conclusion, solitary juvenile polyps in the rectosigmoid area are the most common type of polyps in children. Nevertheless, multiple and proximally located polyps are also present in some pediatric patients. Hematochezia is the main clinical manifestation. The presence of colorectal polyps in children is associated with age ≤ 6 years, positive family aggregation, positive sIgE, higher intake of meat, and lower intake of vegetable.

## Data Availability

The datasets used and/or analyzed during the current study are available from the corresponding author on reasonable request.
